# The Role of T‐Helper 17 Cells and Regulatory T Cells in Acute Diffuse and Total Alopecia: The Increased Function of Regulatory T Cells May Explain the Favorable Prognosis

**DOI:** 10.1111/1346-8138.17745

**Published:** 2025-05-24

**Authors:** Ji‐Hoon Lim, Da‐Hyun Kang, Haena Moon, Soon‐Hyo Kwon, Bark‐Lynn Lew

**Affiliations:** ^1^ Department of Dermatology, Kyung Hee University hospital at Gang‐Dong Kyung Hee University School of Medicine Seoul Korea

**Keywords:** alopecia areata, cytokines, pathogenesis, regulatory T cell, T‐helper 17 cell

## Abstract

**Background:**

Acute diffuse and total alopecia (ADTA) is a variant of alopecia areata (AA) that lacks the typical patchy hair loss seen in classical AA and presents with an acute onset of diffuse hair loss. It has a favorable prognosis.

**Objectives:**

The purpose of this study was to assess the role of T‐helper (Th) 17 cells and regulatory T cells (Tregs) in the pathogenesis of ADTA and their relevance to the good prognosis of ADTA.

**Methods:**

Twenty‐four patients with ADTA and 12 healthy controls were included. Scalp skin samples were obtained for quantitative polymerase chain reaction (qPCR) specific for Th17 cells and Treg‐related cytokines. Serum cytokines associated with Th17 cells and Tregs were measured using enzyme‐linked immunosorbent assays. Additionally, we performed immunostaining for Th17 cells and Tregs. The correlation of qPCR results, serum cytokine levels, and immunostaining results with clinical characteristics was examined.

**Results:**

Lesional IL‐2, IL‐10, and IL‐23A levels were significantly higher in patients with ADTA than in controls. In the progressive stage, lesional IL‐2, IL‐13, and IL‐23A levels were significantly increased compared to those in controls. Serum IL‐15 levels were significantly lower in patients than in controls in the progressive stage. In the recovery stage, lesional IL‐13 and IL‐23A levels were significantly increased compared to those in controls. The ratios of Th17/CD4+ cells and Tregs/CD4+ cells surrounding hair follicles of the patients were 40.73% and 8.50%, respectively, according to immunostaining results.

**Conclusions:**

The increased activity of Treg cells, suggested through IL‐10 and IL‐15, is a characteristic of ADTA that is distinct from AA. The increased function of Tregs may explain the favorable prognosis of ADTA. Follow‐up studies focusing on Tregs, IL‐10, and IL‐15 are required.

## 1 Introduction

Acute diffuse and total alopecia (ADTA) is a variant of alopecia areata (AA) that lacks the typical patchy hair loss observed in classical AA. In patients with ADTA, most scalp hair sheds easily in the early stages following only a gentle pull. After the onset of abrupt diffuse hair shedding, hair loss rapidly progresses to total baldness within 3–20 weeks [1]. Unlike other subtypes, such as alopecia totalis, ADTA is known to have a relatively good prognosis. Therefore, ADTA does not require aggressive treatment compared to the extensive type of AA. Factors more suggestive of ADTA are approximately 20–30 years of age, female sex, no previous history of AA, rapid progression to total hair loss, and histopathologic findings of pigment incontinence and eosinophilic infiltration [1]. However, the mechanism of rapid hair loss and rapid recovery in ADTA is not known. Why hair falls out quickly and recovers quickly in this disease has not been identified. Therefore, studies on pathogenesis different from that of classic AA are needed in ADTA. Additionally, it is necessary to identify additional markers that suggest ADTA.

AA is an organ‐specific autoimmune disease that is characterized by T‐cell infiltrates and cytokine production around anagen‐stage hair follicles [2, 3]. Although several studies have described the role of T helper (Th) 1 and Th2 cytokines in AA, their role in the pathogenesis of AA has not been fully established. Recent studies have highlighted the relationship between AA, Th17 cells, and regulatory T (Treg) cells. Tregs are essential for the maintenance of immune homeostasis. They are critical in the induction and the maintenance of tolerance to self‐antigens and prevention of autoimmunity by producing TGF‐β and IL‐10 [4, 5]. Activated Th17 cells produce a unique subset of cytokines, IL‐17A, IL‐17F, and IL‐22, which classically induce tissue inflammation and play important role in clearing pathogens during a host defense reaction. The maturation of Th17 cells requires the combined stimulation of naive T cells by both the regulatory cytokine TGF‐β and either IL‐6 or IL‐21 [6, 7]. Elevated numbers of Th17 cells and diminished numbers of Tregs have been documented in patients with rheumatoid arthritis (RA), psoriasis, systemic sclerosis, and other disorders [8, 9, 10, 11].

Our group previously demonstrated that an IL‐17RA polymorphism might contribute to the increased susceptibility to AA [12]. We also reported increased levels of Th17 cytokines in both the lesion and serum of patients with AA compared to controls [13]. Han, et al. reported that, in both the scalp tissue and peripheral blood, Th17 numbers were significantly higher in AA patients, whereas Treg numbers were lower [14]. However, there are insufficient data on ADTA.

In this study, we evaluated the patterns of Th17 and Treg subsets in scalp lesions and peripheral blood of patients with ADTA, correlating outcomes with disease characteristics.

## 2 Methods

### 2.1 Patients and Controls

Twenty‐four patients with ADTA were enrolled between July 2018 and July 2020 at the Department of Dermatology, Kyung Hee University Hospital at Gangdong, Seoul, Korea. ADTA was diagnosed based on physical examination, clinical features, and histopathological findings. Patients who presented with acute and diffuse hair loss at the time of their visit and subsequently experienced spontaneous hair regrowth without any specific treatment during follow‐up were included in the study. Age, sex, disease duration, history of AA, presence of other autoimmune diseases, atopic dermatitis, and family history of AA were recorded. The study protocol was approved by the Institutional Review Board (IRB file No. 2017–08–004‐012). All patients were fully informed about the clinical and laboratory procedures and the purpose of the study and provided informed consent. Controls were healthy people who had neither systemic disease nor scalp disease.

### 2.2 Standard Evaluation

Baseline severity, clinical stage, and immunohistochemical (IHC) staining were evaluated. Baseline severity was evaluated according to the “Severity of Alopecia Tool” score [15]. Patients were divided into five groups based on the extent of alopecia (S1, < 25% hair loss; S2,25%–49% hair loss; S3,50%–74% hair loss; S4,75%–99% hair loss; S5,100% hair loss).

Three clinical stages were defined by two dermatologists considering various factors. First, the initial stage was determined by considering both recent onset (< 2 weeks) and lesser extent (<S2) of alopecia. Second, the progressive stage was determined by longer disease duration (≥ 2 weeks) or wide extent (≥S2) of alopecia or persistent increase in lesion size. Third, the recovery stage was determined considering hair growth at the time of the visit and no evidence of active hair loss.

Quantitative evaluation was performed by analyzing the proportion of positively stained lymphocytes in five randomly selected high‐power fields (×400 magnification) around hair follicles by two independent investigators from Kyung Hee University Hospital at Gangdong observers who were blinded to the clinical data of the subjects.

### 2.3 Experimental Procedures

Scalp biopsies from patients with ADTA were examined along with scalp biopsies from healthy controls. Four‐millimeter and two‐millimeter punch biopsy were obtained from the scalp of each participant. It was obtained from an area with severe hair shedding, including hair follicles, based on the investigator's judgment. The four‐millimeter specimen was used for IHC staining of Th17 and Treg cells. The two‐millimeter specimen was used for real‐time quantitative PCR. Total RNA was extracted from scalp skin using QIAzol Lysis Reagent (Qiagen) and converted to cDNA using the ReverTraAce qPCR RT kit (Toyobo). The cDNA was used as template for qRT–PCR using the TaqMan Gene Expression master mix (life technologies) with the given primers. qRT–PCR conditions were 10 min at 95°C, followed by 40 cycles of 95°C for 15 s and annealing/extension for 60 s at 60°C. The amplification was performed on a PCR Step one plus real‐time PCR system (life technologies) and analyzed using the StepOne software v2.3. The relative fold‐change in mRNA expression was calculated using the 2^−△△C^_t_ method. Serum samples from patients and normal controls were collected for performing using ELISA. Serum was centrifuged from 5 mL of blood samples and stored at −80°C until use. The cytokine levels were measured by ELISA kit (all from R&D systems following the protocol recommended by the manufacturer). Scalp biopsies and serum samples were all collected on the same day, at the same clinical stage for each patient.

### 2.4 Statistical Analysis

The Mann–Whitney *U*‐test and Kruskal–Wallis test were used to compare the mean cytokine levels between the patient and control groups as well as between the AA subgroups. The Mann–Whitney *U*‐test was also used to compare the ratio of Th17 and Treg cells between the patients and controls. The chi‐squared test or Fisher's exact test was used to analyze categorical data. Data were analyzed using the Statistical Package for Social Science (SPSS) version 20.0 software (SPSS Inc., Chicago, Il, USA). For all statistical analyses, a 95% level of confidence (*p* < 0.05) was considered statistically significant.

## 3 Results

### 3.1 Baseline Characteristics

Twenty‐four patients with ADTA and 12 healthy controls were analyzed. The mean age of the patients was 38.1 years and the mean age of the controls was 39.0 years. There were eight males (33.3%) in the patient group and six males (50.0%) in the control group. The mean duration was 10.8 weeks. The most prevalent SALT score was S2. Four patients had a history of AA, and three patients had a history of atopic dermatitis (Table 1). Six patients were classified as initial stage, 13 patients were classified as progressive stage, and five patients were classified as recovery stage.

**TABLE 1:** Baseline characteristics.

	Patient (*n* = 24)	Control (*n* = 12)	*p*
Age (Mean ± SD)	38.1 ± 12.6	39.0 ± 20.3	0.8183
Sex			
Male (%)	8 (33.3)	6 (50.0)	0.1399
Female (%)	16 (66.7)	6 (50.0)	
Disease duration (Mean ± SD)	10.8 ± 9.3 weeks		
SALT (%)			
S1	5 (20.8)		
S2	8 (33.3)		
S3	4 (16.7)		
S4	5 (20.8)		
S5	2 (8.3)		
Clinical stage (%)			
Initial	6 (25.0)		
Progressive	13 (54.2)		
Recovery	5 (20.8)		
Nail involvement (%)	6 (25.0)		
AA history (%)	4 (16.7)		
Family history (%)	0 (0.0)		
Atopic dermatitis history (%)	3 (12.5)		

Abbreviations: AA, alopecia areata; SALT, Severity of Alopecia Tool; SD, standard deviation.

### 3.2 Lesional Cytokine mRNA Levels in Scalp Skin

**FIGURE 1:**
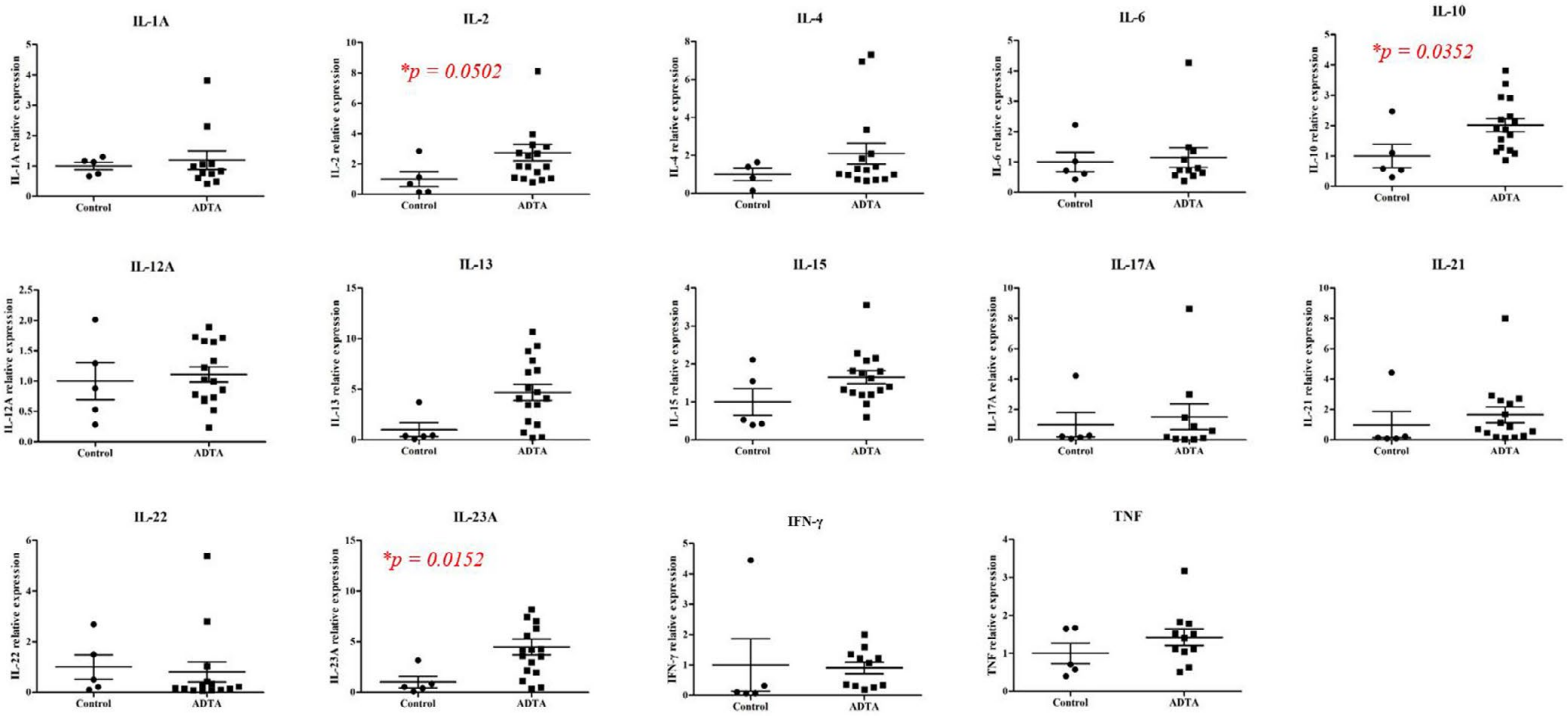
Distribution of lesional cytokine mRNA levels.

Table 2 shows the mean values of lesional cytokine mRNA levels in the scalp skin of the patients and controls. There was a significant increase in IL‐2, IL‐10, and IL‐23A levels in patients compared with controls (Figure 1).

**TABLE 2:** Mean values of lesional cytokine mRNA levels (Relative gene expression).

	IL1A	IL2*	IL4	IL6	IL10*	IL12A	IL13	IL15	IL17A	IL21	IL22	IL23A*	IFNγ	TNFα
Control	1.000 ± 0.248	1.000 ± 0.996	1.000 ± 0.573	1.000 ± 0.639	1.000 ± 0.779	1.000 ± 0.609	1.000 ± 1.359	1.000 ± 0.701	1.000 ± 1.613	1.000 ± 1.719	1.000 ± 0.974	1.000 ± 1.107	1.000 ± 1.726	1.000 ± 0.547
ADTA	1.193 ± 0.304	2.749 ± 0.548	2.094 ± 0.552	1.149 ± 0.329	2.019 ± 0.218	1.108 ± 0.126	4.687 ± 0.791	1.647 ± 0.165	1.514 ± 0.815	1.656 ± 0.523	0.802 ± 0.372	4.488 ± 0.767	0.901 ± 0.191	1.425 ± 0.217

*Note:* The Mann–Whitney *U*‐test was used.

Abbreviation: ADTA, acute diffuse and total alopecia.

* *p* value < 0.05.

In the analysis conducted according to the clinical stage of ADTA, there were significant increases in IL‐2, IL‐13, and IL‐23A in the patient group with progressive disease compared with controls. Furthermore, there was a significant increase in IL‐13 and IL23A levels in the group of patients in the recovery group compared with the controls (Table 3).

**TABLE 3:** Lesional cytokine analysis according to clinical stages.

		IL‐1A	IL‐2	IL‐4	IL‐6	IL‐10	IL‐12A	IL‐13	IL‐15	IL‐17A	IL‐21	IL‐22	IL‐23A	IFNγ	TNFα
Initial stage (*n* = 6)	Mean	3.0655	2.0186	2.7155	0.6488	2.3079	1.0019	2.8128	1.4881	2.2473	1.6359	0.2469	2.7924	0.7692	1.2800
	SD	0.7613	0.6605	2.7094	0.0829	1.1158	0.5267	1.5123	0.2466	0.7667	1.1251	0.1474	1.4838	0.4599	0.2359
	*p*	0.095	0.286	0.686	0.857	0.063	0.905	0.413	0.413	0.381	0.190	0.393	0.286	0.571	1.000
Progress stage (*n* = 13)	Mean	0.8030	3.4928	2.0538	1.6012	2.0127	1.1119	5.3117	1.9557	1.6155	1.9720	0.9394	5.2670	0.9103	1.6173
	SD	0.2166	2.7372	2.0175	1.2305	0.8380	0.4059	3.9288	0.7582	3.1495	2.5908	1.7027	3.8331	0.6635	0.7911
	*p*	0.247	**0.042** *	0.683	0.247	0.073	0.755	**0.042** *	0.106	0.792	0.202	0.524	**0.029** *	0.177	0.329
Recovery stage (*n* = 6)	Mean	0.5961	2.1372	1.4091	0.5929	1.7612	1.0585	5.3022	1.4385	0.4776	1.4403	1.4606	4.4857	1.3251	1.4505
	SD	0.3818	0.4010	3.7459	38.9547	1.3666	1.2674	1.2573	0.2929	0.0413	0.0423	105.511	11.3253	0.1666	0.0033
	*p*	0.381	0.250	0.533	0.571	0.286	1.000	**0.036** *	0.413	0.857	0.250	1.000	**0.036** *	0.381	0.381
Control (*n* = 12)	Mean	1.0000	1.0000	1.0000	1.0000	1.0000	1.0000	1.0000	1.0000	1.0000	1.0000	1.0000	1.0000	1.0000	1.0000
	SD	0.2481	0.9955	0.5733	0.6392	0.7788	0.6093	1.3592	0.7009	1.6131	1.7186	0.9740	1.1069	1.7256	0.5465

*Note:* The *p* values in the table are comparisons between each stage and the control group. The Mann–Whitney *U*‐test was used.

Abbreviation: SD, standard deviation.

* *p* value < 0.05.

There were no differences between the groups in terms of age, disease duration, severity, nail involvement, history of AA, and family history of AA.

### 3.3 Serum Cytokine Levels in Venous Blood

There was no significant difference between the mean serum cytokine levels in the blood samples of the patients and those of controls. However, in association with clinical stage, there was a significant decrease in IL‐15 in patients with progressive disease compared to controls (Table 4). In addition, the decrease in IL‐15 levels in patients at the progressive phase was also significant when compared to those on patients at the recovery stage (*p* = 0.031).

**TABLE 4:** Serum cytokine analysis according to clinical stages.

		IL‐1β	IL‐2	IL‐4	IL‐6	IL‐10	IL‐12	IL‐13	IL‐15	IL‐17	IL‐22	IL‐23	IFN‐γ	TNFα	TGFα	TGFβ
Initial stage (*n* = 6)	Mean	0.1072	0.1516	0.0345	1.7098	0.2712	0.2052	255.9714	1.7228	0.6373	30.1458	68.5313	0.9229	1.0037	37.2950	4.6206
	SD	0.0860	0.1141	0.0012	1.6912	0.1743	0.1593	70.2297	0.6511	0.2297	15.6064	12.4247	0.4769	0.3185	9.7217	5.2300
	*p*	0.200	1.000	0.841	0.548	0.400	0.667	0.381	1.000	1.000	0.421	0.571	1.000	0.548	1.000	0.905
Progress stage (*n* = 13)	Mean	0.1216	0.1382	0.0356	1.2655	1.2535	0.2391	298.5000	1.0658	0.4462	24.1989	55.2280	1.0355	0.8692	67.1667	3.1196
	SD	0.1666	0.0423	0.0033	1.2674	1.3666	0.2929	105.5116	0.4010	0.0413	3.8993	11.3253	1.2573	0.3818	38.9547	3.7459
	*p*	0.667	0.594	0.768	0.797	0.230	1.000	0.711	**0.019** *	0.091	0.438	0.161	0.304	0.310	0.298	0.797
Recovery stage (*n* = 6)	Mean	0.7890	0.1959	0.0350	1.2616	0.1555	0.2635	288.0500	1.5850	0.6351	38.1433	69.4667	1.5704	0.8652	62.4820	8.0992
	SD	1.2896	0.0513	0.0038	0.5487	0.1110	0.1488	52.4542	0.2426	0.2899	17.3899	6.8505	2.0317	0.3264	28.6955	5.1269
	*p*	0.393	0.177	0.792	0.792	1.000	1.000	0.643	1.000	1.000	0.177	1.000	0.762	0.429	0.151	0.537
Control (*n* = 12)	Mean	0.0400	0.1320	0.0341	1.2205	0.2137	0.2194	329.9000	1.5956	0.7205	25.8936	72.0000	1.0292	1.0941	36.1000	6.3573
	SD	0.0280	0.0699	0.0005	0.7250	0.2129	0.1616	67.9000	0.2045	0.1498	16.1953	10.1116	0.6103	0.3351	10.2395	6.2207

*Note:* The *p* values in the table are comparisons between each stage and the control group. The Mann–Whitney *U*‐test was used.

* *p* value < 0.05.

There were no differences between the groups in terms of age, disease duration, severity, nail involvement, history of AA, and family history of AA.

### 3.4 Th17 and Treg Ratio in Scalp Skin

**FIGURE 2:**
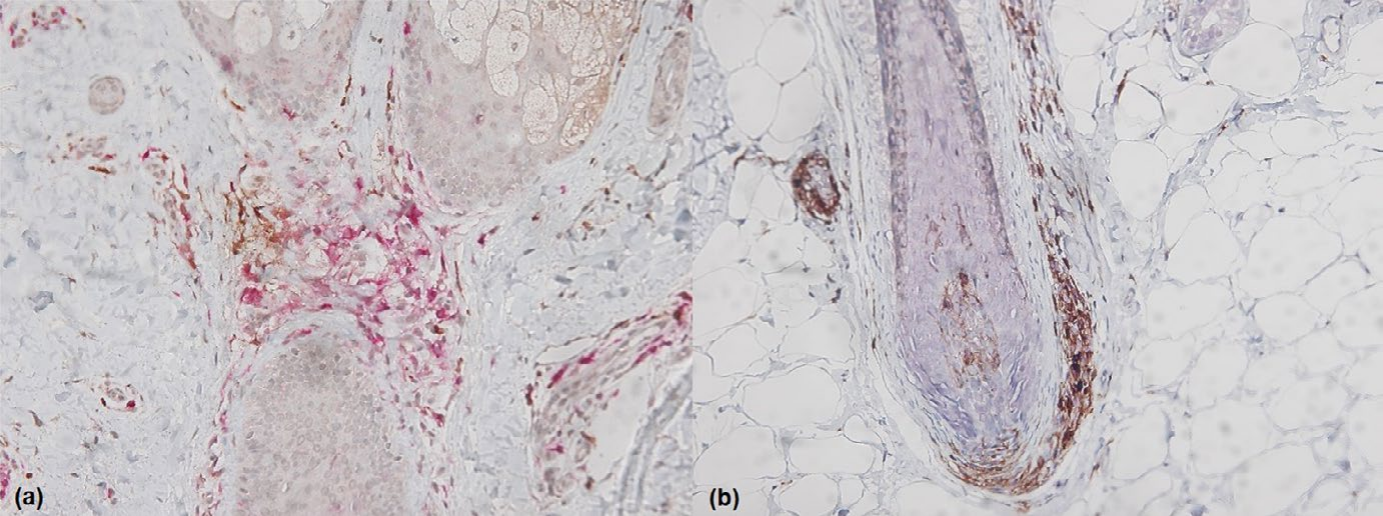
(×100) IHC staining for CD4, IL‐17, FoxP3, and CD25 to visualize Th17 and Treg cells in scalp lesions. (a) IL‐17(DAB) and CD4(red) stain for Th17 cells (IL‐17^+^CD4^+^ cells) (b) FoxP3(blue), CD25(red), and CD4(DAB) stain for Treg cells (FoxP3^+^CD25^+^CD4^+^ cells).

To visualize Th17 and Treg cells in scalp lesions, IHC staining for CD4, IL‐17, FoxP3, and CD25 was performed (Figure 2). The numbers of Th17 cells (IL‐17^+^CD4^+^ cells) surrounding the hair follicles of AA patients were significantly higher than those in control tissues. Conversely, the numbers of Treg cells (FoxP3^+^CD25^+^CD4^+^ cells) around the hair follicles of patients, expressed as the proportion of positive lymphocytes, were significantly decreased compared with those in control tissues (Table 5).

**TABLE 5:** Ratio of Th17 and Treg cells in patients and controls.

	Patients	Controls	*p*
Th17 (CD4^+^IL17^+^)/CD4^+^ cells	40.73% ± 14.67%	23.64% ± 12.38%	0.003*
Treg (CD4^+^FoxP3^+^CD25^+^)/CD4^+^ cells	8.50% ± 5.21%	12.61% ± 7.39%	0.039*

*Note:* The Mann–Whitney *U*‐test was used.

Abbreviations: Th, T‐helper; Treg, regulatory T.

* *p* value < 0.05.

## 4 Discussion

In this study, we found a difference in the lesional and serum cytokine profiles between patients with ADTA and healthy controls. Various changes were observed in cytokines related to Th1 (IL‐2, IL‐15), Th2 (IL‐13), Th17 (IL‐23), and Treg (IL‐10) cells. Lesional IL‐2, IL‐10, and IL‐23A levels were significantly higher in patients with ADTA than in controls. In patients at the progressive stage, lesional IL‐2, IL‐13, and IL‐23A levels were significantly increased, and serum IL‐15 was significantly decreased compared to controls. In patients with ADTA at the recovery stage, lesional IL‐13 and IL‐23A levels were significantly higher than in controls.

The Th1 cytokine IL‐2 is a lymphokine produced by activated T cells and the IL‐2 receptor is expressed on T cells. IL‐2/IL‐2R have been implicated in the pathogenesis of AA. Several studies have reported significantly elevated lesional and serum levels of IL‐2 in patients with AA compared with controls [16, 17, 18]. The results of these reports are consistent with our findings of increased IL‐2 levels in ADTA lesions.

IL‐23 is essential for the differentiation of Th17 lymphocytes and activates JAK2 and TYK2 in human keratinocytes [19]. Furthermore, IL‐23 drives inflammation and hyperplasia in mouse skin independently of IL‐17A [20]. Thus, IL‐23 can affect follicular growth in an IL‐17/IL‐22–independent manner. A study reported an increase in lesional IL‐23 in patients with AA compared to healthy controls [21]. Our group previously demonstrated an increase in IL‐23 in both the serum and lesion of patients with AA compared to normal controls [13]. This study on ADTA also showed comparable results. As the relationship between Th17 and AA has recently been highlighted [14], it has been confirmed that Th17 also plays a role in ADTA. The increased Th17 cell infiltration around hair follicles, confirmed by IHC staining in this study, also supports this hypothesis. However, IL‐17, a key cytokine of Th17 cells, showed no significant difference between patients with ADTA and controls. Previous studies have confirmed the increase in serum and lesional IL‐17 levels in patients with AA compared to controls [13, 22, 23]. Moreover, we previously demonstrated for the first time that IL‐17RA polymorphisms might contribute to the increased susceptibility of Koreans to AA [12]. Intriguingly, two studies reported decreased expression of IL‐17 in ADTA compared with other subtypes of AA [13, 23]. This study did not show a significant effect on IL‐17 compared with the control group. Therefore, it can be inferred that Th17 cells in ADTA are less active than those in classic AA.

IL‐13 is also involved in Th2‐mediated inflammation. Previous studies have shown a significant increase in lesional and serum IL‐13 levels in patients with AA compared to healthy controls [13, 16, 21]. These findings are consistent with those of our study on ADTA. As in AA, Th2 cells appear to play a role in ADTA to some extent.

IL‐15 signaling is known to evoke a Th1‐type immune response by inducing the release of IFN‐γ and TNF‐α [24]. In addition, IL‐15 limits the suppressive effect of Treg cells [25, 26]. Previous studies on AA have shown that lesional IL‐15 is increased in patients compared with normal controls [21, 27]. IL‐15 plays a key role in inducing hair follicle‐associated CD8+ T cells, which are important cells in AA pathogenesis, and maintaining them there. IL‐15 and its receptors on CD8+ T cells showed increased expression around HFs, and blocking IL‐15 prevented AA in mice [18, 27, 28]. In contrast, in our study, serum IL‐15 levels were significantly decreased in patients at the progressive stage compared with the controls and patients at the recovery stage. Considering that a decrease in IL‐15 induces the activation of Treg cells and inhibits the induction of CD8+ T cells in the hair follicle area, this is a characteristic result of this study. However, lesional IL‐15 levels did not show significant differences. This may be attributed to the influence of other cytokines associated with activated Treg cells, but further large‐scale studies are needed to explore this aspect in more detail.

Tregs are essential for the maintenance of immune homeostasis. They are critical in the induction and maintenance of tolerance to self‐antigens and the prevention of autoimmunity by producing IL‐10 [4]. A few studies have demonstrated the role of Treg cytokines in AA, but the results are conflicting. Most studies showed no significant differences in Treg cytokines between patients with AA and controls [5, 17, 29]. However, our study demonstrated an increased expression of lesional IL‐10 in patients with ADTA compared to controls. Together with IL‐15, this study implied that Treg‐related cytokines are characteristically expressed in ADTA. Staining also confirmed that the ratio of Treg cells in this study was higher (8.50%) than that in the previous study on AA [14] (6.32%), although lower than that in the control group. This comparison is not meaningful from a methodological point of view, but provides a clue to the role of Tregs in ADTA.

The characteristic of ADTA is that hair loss progresses faster and recovers faster than in classic AA. Through this study, it appears that ADTA is characterized by increased Treg activation and decreased Th17 activation compared with the characteristics of AA identified in previous studies. These differences may have contributed to the rapid recovery from ADTA. We suggest that the active expression of Tregs in ADTA competes with the immune response, which is involved in the progression of AA. The predominance of the Treg response may be a contributing factor to the rapid recovery of this disease.

The limitation of our study is that it was conducted at a single institution with a small sample size. Additionally, we did not directly compare the lesional and serum profiles of cytokines or T‐cell subsets between ADTA and other types of AA. As a result, the conclusions of this study are not fully justified based solely on its findings but are largely supported by our previous studies on AA. Therefore, further research with a larger cohort is needed to directly compare ADTA and AA.

In conclusion, in this study, the increased numbers of Treg cells, suggested through increased lesional IL‐10 and decreased serum IL‐15 levels and higher ratio, is a characteristic of ADTA that is distinct from the characteristics of AA identified in previous studies. The increased function of Tregs may explain the favorable prognosis of ADTA. Follow‐up studies focusing on Tregs, IL‐10, and IL‐15 are required.

## Conflicts of Interest

The authors declare no conflicts of interest.
